# The impact of low back pain and vigorous activity on mental and physical health outcomes in older adults with arthritis

**DOI:** 10.3389/fpain.2022.886985

**Published:** 2022-07-22

**Authors:** Janiece L. Taylor, Natalie G. Regier, Qiwei Li, Minhui Liu, Sarah L. Szanton, Richard L. Skolasky

**Affiliations:** ^1^Johns Hopkins School of Nursing, Baltimore, MD, United States; ^2^Johns Hopkins School of Nursing Center for Innovative Care in Aging, Baltimore, MD, United States; ^3^Xiangya School of Nursing, Central South University, Changsha, China; ^4^Department of Orthopaedic Surgery, Johns Hopkins University School of Medicine, Baltimore, MD, United States

**Keywords:** low back pain, disability, physical activity, older adults, depressive symptoms

## Abstract

**Background:**

Nearly 50% of Americans aged 65 and above have been diagnosed with arthritis and an estimated 80% of adults experience low back pain (LBP). Little is known about the experience of LBP in older adults with arthritis and its relationships with mental and physical health.

**Objective:**

In this study, we examined the relationships between LBP and four physical and mental health conditions (psychological distress, insomnia, mobility limitations, and self-rated health) in older adults with arthritis in the National Health and Aging Trends Study (NHATS). We also examined whether vigorous exercise mediated the relationships between LBP and these four conditions.

**Materials and Methods:**

The data from this study comes from waves five through nine of the NHATS. The sample size ranged from 3,490 to 2,026 across these waves. All variables in this study are based on self-report. We used descriptive analyses including means and standard deviations for continuous variables or frequencies and proportions for demographic data. We used structural equation modeling (SEM) to examine if vigorous activity mediated the relationship between LBP with the four conditions.

**Results:**

The age range of the sample was 65 years of age and older. Among those with back pain 78.53% had no mobility limitations. There was a significant relationship between LBP with insomnia (*B* = 0.48, *p* < 0.001), perceived health status (*B* = −0.38, *p* < 0.0010), and psychological distress (0.67, *p* < 0.001). Activity mediated the relationship between LBP and insomnia, psychological distress and physical health in adjusted models.

**Discussion:**

The presence of low back pain in older adults with arthritis increases the risk of insomnia, psychological distress, mobility limitations, and poorer self-rated health. Consequently, targeting comorbid LBP may be an important component of the treatment plans of older adults with arthritis. In addition, providers of patients with arthritis and LBP should conduct routine assessments of mental and physical health to ensure the LBP is being adequately addressed.

## Introduction

Low back pain (LBP) is the single leading cause of disability worldwide, affecting ~540 million people at any given time (1). In the United States, back pain is the most common type of musculoskeletal pain reported by older adults and an estimated 80% of adults experience low back pain (LBP) at some point in their lives (2). In addition to disability, it is well-documented that pain, especially musculoskeletal-related pain, is associated with limitations in mobility, worsened mental health, insomnia, and poorer self-rated health (3–7).

LBP, chronic or otherwise, is commonly associated with musculoskeletal comorbidities such as osteoarthritis (OA), rheumatoid arthritis, and osteoporosis, and various factors exacerbate LBP irrespective of the associated musculoskeletal comorbidity (8–11). The interaction of OA (e.g., of the hip, knee, and spine) with chronic LBP contributes to increased disability, possibly due to exacerbation of pain (12–19).

Among the common suggested treatments for LBP is vigorous activity; however, little is known about overall effectiveness of vigorous activity for treating pain and related symptoms. Findings are mixed regarding exercise and outcomes typically associated with LBP (20–25). In young- and- middle-aged adults, vigorous activity moderate to high levels of exercise/activity has been shown to buffer insomnia related to back pain (25). In one study, researchers reported that cycling reduced the risk of LBP over time (20, 24). Researchers have examined whether exercise is related to mental health outcomes, sleep and disability in individuals with LBP (22–24); however, few researchers have looked at exercise as the potential mediator between LBP and these associated conditions. Further understanding the effects of vigorous activity on common LBP and related conditions in older adults may help identify if similar buffering effects occur in older populations. In addition, the benefits of physical activity in older adults with LBP may have positive spillover effects onto mental and physical health conditions related to back pain.

The present study is grounded in the biopsychosocial model of pain (26). This model asserts that, although there is a physiological response to pain, the way in which a person experiences or perceives pain is based on multiple factors including biological or genetic composition, prior learning, and sociocultural and psychological influences (26). Per Wachholtz et al. (27), “This model facilitates an approach to the treatment of medical illness and pain that recognizes the complex interactions between individuals and their environment, as well as the potential bidirectional pathways between psychological and biological factors with regard to disease.” Regarding physical factors, LBP is known to be a key contributor to the global burden of disability. Vigorous activity has been shown to counteract pain catastrophizing, strengthen muscles and bones, help people with pain return to adaptive activity, and ultimately reduce pain in some circumstances (28–30). Hence, we conceptualized physical activity as a potential mediator in our analyses. Specifically, we examine vigorous activity as a hypothesized mediator, which may be related to psychological and psychosocial outcomes. Regarding variables we identified variables from the physiological, psychosocial, and biological domains that are related to LBP. Insomnia has been identified as a CNS pain generator in persons with chronic low back pain (31), and as an it is classified as a psychophysiological disorder, and we also felt it fit within the psychological component of the biopsychosocial framework as an evidence-based outcome variable in our model. Mobility limitations are often the body's response to various conditions and has been linked to LBP. We classify this as a physiological response to pain (32). Self-rated health has been identified as a psychosocial factor related to LBP and the prognosis of LBP in previous work (33, 34). We categorized self-rated health as a psychosocial factor associated with LBP for this study.

Identifying conditions related to LBP in older adults may elucidate how they are experiencing or living with their pain. A better understanding of consequences specific to LBP in older adults can assist with intervention development that will address not just LBP but potentially associated conditions as well.

This study examines the relationship of LBP with mobility limitations, psychological distress, insomnia, and self-rated health status in order to more clearly define the burden experienced by older adults with arthritis over a 6-year period. We specifically examine if vigorous activities are mediating factors within the biopsychosocial model of pain. We hypothesize that (1) LBP will be independently associated with more mobility limitations, insomnia, psychological distress, and poor self-rated health, and (2) vigorous activity will mediate the relationship between LBP with mobility limitations, insomnia, psychological distress, and poor self-rated health.

## Methods

### Procedures and data collection

Data for this study were collected as part of the National Health and Aging Trends Study (NHATS), a nationally representative sample of Medicare beneficiaries ages 65 and older. The sampling frame included all persons enrolled in Medicare, representing 96% of U.S. Medicare beneficiaries, and excluded 4% of older adults ineligible for Medicare for various reasons (27). A stratified, three-stage design was implemented for participant selection, first drawing from counties/groups of counties, then ZIP codes, and lastly from Medicare beneficiaries enrolled as of September 30, 2010 (35). The NHATS is an ongoing study with data collection occurring on an annual basis beginning in 2011. Measures collected include information on participants' physical capabilities, economic status, cognition, living arrangements, wellbeing, and activity engagement, among other variables. More detailed information related to the NHATS design and recruitment procedures is available elsewhere (35, 36). We included NHATS participants from waves 5–9 (2015–2019) who were living in the community and reported having arthritis at the time of data collection. The inclusion criteria were: (1) age 65 and above, (2) community-dwelling, and (3) comorbid diagnoses of arthritis and low back pain.

### Instruments

#### Pain

Back, knee, and hip pain were measured by a yes/no question asking if respondents had been bothered by pain in the last month, and a follow-up question asking participants to specify the site(s) of pain.

#### Mobility limitations

Participants were asked if they had difficulties or needed help with going outside, getting around outside, or getting in and/or out of bed. For the demographics we examined the number of mobility limitations. For the mediation analysis a binary mobility limitation variable was created that indicated difficulties or no difficulties with mobility. Each mobility item was binary coded and a total number was calculated to represent the number of mobility limitations a participant had.

#### Psychological distress

Participants were asked “over the last month, have you ever felt down/depressed/anxious?” The scores of these two items were averaged to represent the severity of depression (range from 0 to 6). In accordance with the PHQ-2 criteria, participants were classified as having depressive symptoms if the cumulative score for both items was 3 or more (37).

#### Insomnia

Difficulty initiating sleep and difficulty maintaining sleep were determined by asking the questions “How often does it take you more than 30 min to fall asleep at night?” and “How often do you have trouble falling back to sleep on nights after waking up from sleep?” Responses to these questions included 0 (*never*), 1 (*rarely*), 2 (*some nights*), 3(*most nights*), and 4 (*every night*). The total score ranged from 0 to 8, with a higher total score stands for more insomnia.

#### Health status

Participants' self-rated health was measured by a single question asking them to rate their general health using a Likert scale where 4 indicates “excellent health” and 0 indicates “poor health”. Higher scores indicate worse self-rated health.

#### Vigorous activity

Participants' self-report of participation in physical activities was a dichotomous (yes/no) variable assessed by a single question asking, “In the last month, did you ever spend time on vigorous activities that increased your heart rate and made you breathe harder?” Sample activities included working out, swimming, running, biking, or playing a sport.

#### Potential covariates

We sought to understand the association of LBP with mobility limitations, psychological distress, and insomnia in older adults with arthritis and to determine whether vigorous activity would mediate those associations. Potential covariates were identified by consulting the literature. Fehrmann et al. (38) examined age and gender as moderators on the influence of chronic LBP on activities, participation, and environmental factors. We also adjusted for age, gender, highest level of education, and marital status.

### Statistical analyses

Descriptive analyses, including means and standard deviations for continuous variables or frequencies and proportions for categorical variables, were used to summarize the demographic and functional characteristics of the samples from 2015 to 2019. Independent *t*-tests for continuous variables and chi-square tests for categorical variables were used to compare demographic characteristics and health-related variables for persons with and without self-reported back pain.

This study applied structural equation modeling (SEM) to examine the association of back pain (distinct from other pain) with psychological distress, insomnia, and self-rated health status and random effect logit models to examine the association of back pain with mobility limitations. These models adjusted for potential confounding covariates as previously defined. The main constructs in this study (e.g.., back pain, vigorous activities, and health outcomes) were measured at each point of time. For example, the latent variable pain was measured by back pain status from the fifth to ninth waves. By using latent variables in SEMs, we could also see the impact of each measurement over time.

Across all models, the latent pain variable served as the independent variable, vigorous activities were applied as the mediating variable, and health outcomes were integrated into the SEM model as the dependent variable. In the models, the direct effects from pain to vigorous activities are marked as *a*, the direct effects from vigorous activities to health outcomes are marked as *b*, the direct effects from pain to health outcomes are marked as *c*. The indirect effects are calculated by multiplying *a* and *b* (a^*^b). Missing values in the data set were handled using maximum likelihood estimation. Data management and analyses were all performed using R (ver.4.1.2) and the Laveran package (ver.0.6-9).

## Results

### Sample demographic characteristics

Table 1 provides descriptive characteristics for the total sample of older adults with arthritis and by the presence of LBP. At the baseline wave in 2015, respondents were primarily female (64.54%), White (67.29%), and 48.59% lived with a spouse, partner, or others. Most of the participants engaged in little-to-no vigorous activity on a regular basis (67.57%). White older adults were significantly more likely to report back pain (*p* = 0.021) than non-Whites. Among older adults with LBP, psychological stress scores were higher (*p* < 0.001), insomnia levels were higher (*p* < 0.001), poorer health was reported (*p* < 0.001), and more mobility limitations were reported (*p* < 0.001), compared to older adults without LBP conditions.

**Table 1:** Sample characteristics.

	**Total**		**With back pain**	
	***N***	**%**	***N***	**%**
**Age**				
65–69	1,187	7.61%	602	8.08%
70–74	3,518	22.56%	1,757	23.60%
75–79	3,681	23.60%	1,819	24.43%
80–84	3,292	21.11%	1,531	20.56%
85–89	2,341	15.01%	1,073	14.41%
90+	1,578	10.12%	664	8.92%
**Gender**				
Female	10,067	64.54%	4,935	66.28%
Male	5,530	35.46%	2,511	33.72%
**Race**				
White	10,495	67.29%	5,126	68.84%
Black	3,487	22.36%	1,599	21.47%
Hispanic	371	2.38%	168	2.26%
Other	1,244	7.98%	553	7.43%
**Education**				
Low	3,563	23.23%	1,725	23.54%
Medium	8,321	54.24%	4,117	56.18%
High	3,456	22.53%	1,486	20.28%
**Marital**				
Not married	8,012	51.41%	3,894	52.32%
Married	7,572	48.59%	3,549	47.68%
**Health**				
Poor	1,085	6.96%	713	9.58%
Fair	3,674	23.57%	2,115	28.42%
Good	5,728	36.74%	2,683	36.05%
Very good	3,997	25.64%	1,605	21.56%
Excellent	1,106	7.09%	327	4.39%
**Vigorous activities**				
No	10,532	67.57%	5,198	69.85%
Yes	5,055	32.43%	2,244	30.15%
Depressive symptoms		7,442	
Mean (SD)		1.80 (2.26)		2.28 (2.51)
**Insomnia**				
Mean (SD)		3.04 (2.05)		3.34 (2.06)
**Mobility limitations**				
0	12,652	81.12%	5,847	78.53%
1	1,566	10.04%	826	11.09%
2	762	4.89%	435	5.84%
3	617	3.96%	338	4.54%
Mean (SD)		0.12 (0.50)		0.38 (0.82)

### Mediation of the relationship between back pain and outcome variables

**Figure 1:**
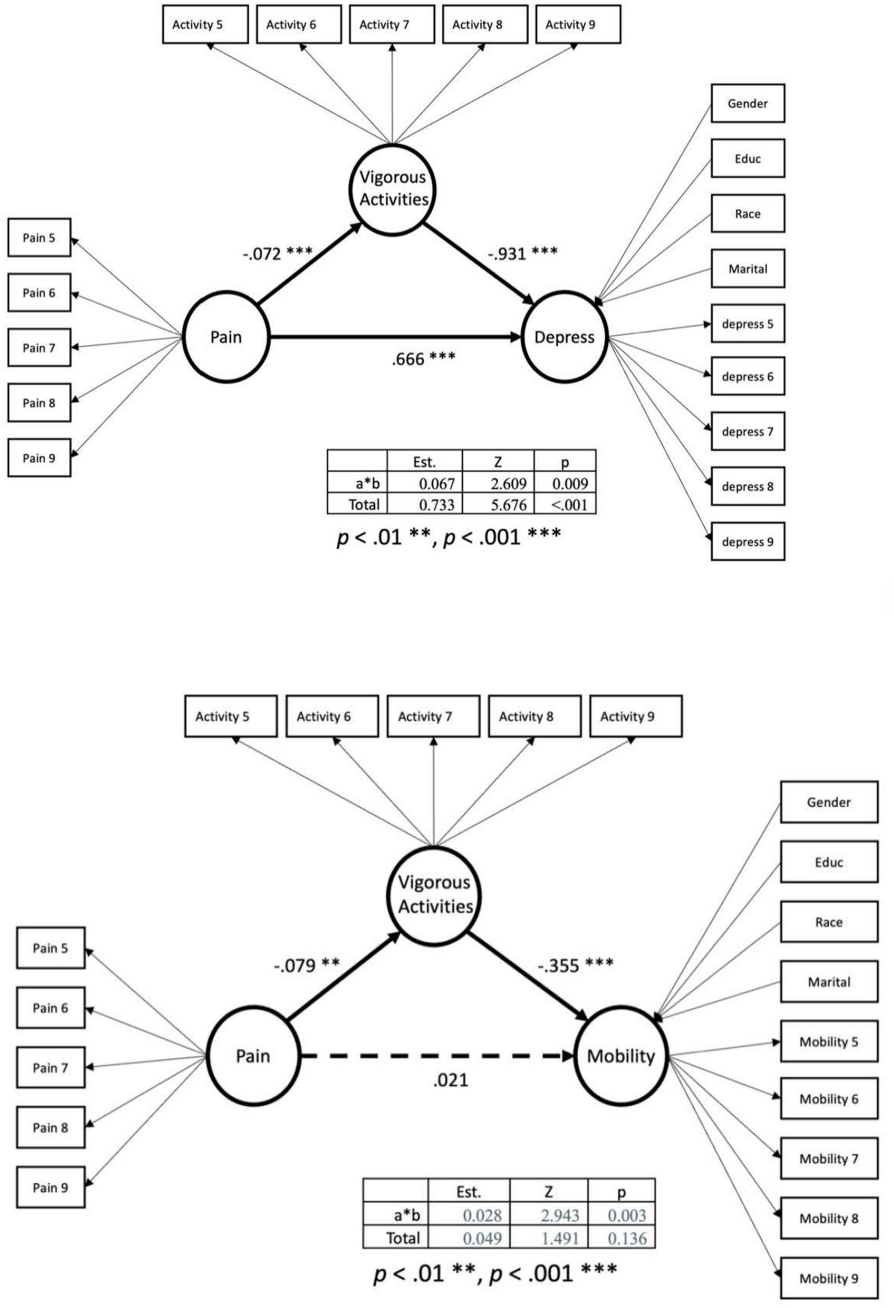
Mediation model with vigorous activities mediating relationship between pain and mobility.

**Figure 2:**
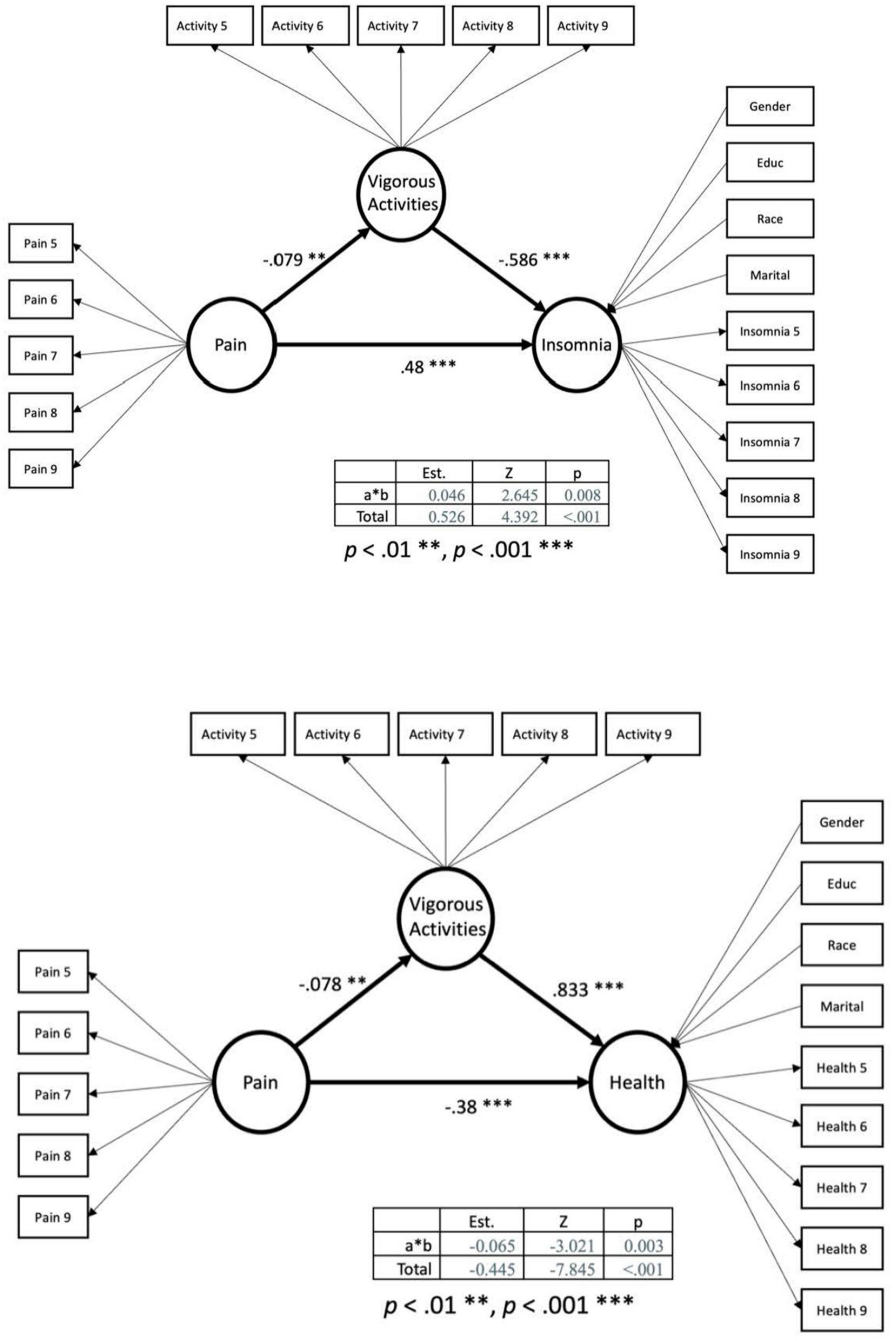
Mediation model with vigorous activities mediating relationship between pain and health.

Table 2 summarizes the results from the mediating models. Table 3 summarizes the model fit for the four mediating models. In Figure 1 the SEM models of the mediation modeling are presented looking at vigorous activities mediating the relationship between pain and depression and pain and mobility. In Figure 2 the SEM models of the mediation modeling are presented looking at vigorous activities mediating the relationship between pain and insomnia and pain and health.

**Table 2:** Summary of the mediating effects of models.

**Variables**		**Estimation**	***Z-*** **value**	***p*** **-value**
Depression	c	0.666	5.216	<0.001
	b	−0.931	−7.291	<0.001
	a	−0.072	−2.751	0.006
	ab	0.067	2.609	0.009
	Total	0.733	5.676	<0.001
Health	c	−0.38	−7.062	<0.001
	b	0.833	14.633	<0.001
	a	−0.078	−3.041	0.002
	ab	−0.065	−3.021	0.003
	Total	−0.445	−7.845	<0.001
Insomnia	c	0.48	4.018	<0.001
	b	−0.586	−5.045	<0.001
	a	−0.079	−3.037	0.002
	ab	0.046	2.645	0.008
	Total	0.526	4.392	<0.001
Mobility	c	0.021	0.65	0.516
	b	−0.355	−10.684	<0.001
	a	−0.079	−3.041	0.002
	ab	0.028	2.943	0.003
	Total	0.049	1.491	0.136

*aThe direct effects from pain to vigorous activities*.

*bThe direct effects from vigorous activities to health outcomes*.

*cThe direct effects from pain to health outcomes*.

abThe indirect effects (a^*^b); and Total means the total effects.

**Table 3:** Model fits of mediating models with the four outcomes.

**Outcomes**	χ^2^	**df**	***p*** **-value**	**CFI**	**TLI**	**RMSEA**
Health	791.683	126	<0.001	0.952	0.944	0.047
Mobility	930.908	126	<0.001	0.937	0.926	0.051
Insomnia	711.014	126	<0.001	0.952	0.943	0.044
Depression	710.592	126	<0.001	0.945	0.936	0.044

*CFI, comparative fit index; TLI, Tucker-Lewis index; RMSEA, the root mean square error of approximation*.

According to Hu and Bentler (39), a good model fit is characterized as having a value close to 0.95 for TLI and CFI and a value smaller than 0.06 for RMSEA. The values of model fit for the mediating model for health were, χ^2^ = 791.683, *df* = 126, *p* < 0.001, CFI = 0.952, TLI = 0.944, and RMSEA = 0.047, indicating that the model is well fit. All loadings from observations to latent variables were statistically significant. Back pain was negatively associated with the perceived health status of community-dwelling older adults with arthritis, *B* = −0.38, *z* = −7.06, *p* < 0.001, indicating that having back pain was related to poorer perceived health status. In addition, back pain was also negatively associated with older adults' level of vigorous activities, *B* = −0.078, *z* = −*3.04, p* = 0.002. Vigorous activities were positively associated with the health status, *B* = 0.833, *z* = *14.63, p* < 0.001. The total effect from back pain to health status was −0.445 and the indirect effect was −0.065, indicating that about 15% of the effect from back pain to the health status of community-dwelling older adults with arthritis was mediated by their levels of vigorous activities.

The model fit of the mediating model for depressive symptoms was a strong model, χ^2^ = 710.592, *df* = 126, *p* < 0.001, CFI = 0.945, TLI = 0.936, and RMSEA = 0.044. Back pain correlated with higher levels of depressive symptoms by 0.67, *z* = 5.22, *p* < 0.001, demonstrating that back pain may increase the depressive symptoms. Additionally, back pain was negatively correlated to the level of vigorous activities, *B* = −0.072, *z* = −*2.75, p* = 0.006. The total effects from back pain to depressive symptoms were 0.733, *p*< *0.001*, and the mediating effect was 0.049, *p* = 0.067. The proportion of mediated effects was 9.14%.

The third model tested the level of insomnia as the outcome variable and demonstrated a strong model fit, χ^2^ = 711.014, *df* = 126, *p* < 0.001, CFI = 0.952, TLI = 0.943, and RMSEA = 0.044. Back pain was still statistically and significantly predictive of the levels of insomnia and vigorous activities, with *B* = 0.48, *z* = *4.02, p* < 0.001, and *B* = −0.078, *z* = −*3.04, p* = 0.002, respectively. Vigorous activities were related to lower levels of insomnia, *B* = −0.586, *z* = −*5.05, p* < 0.001. The indirect effect from back pain to insomnia was 0.046 while the total effects were 0.526, showing a 9% mediating effects.

The model fit was acceptable, χ^2^ = 930.908, *df* = 126, *p* < 0.001, CFI = 0.937, TLI = 0.926, and RMSEA = 0.051. Back pain was not associated with changes in mobility whereas vigorous activities were negatively related to mobility. The total effects from back pain to mobility were not statistically significant.

## Discussion

Vigorous activity may improve back pain (40); hence, we hypothesized that vigorous activity would mediate the relationship between LBP and four health outcomes, i.e., mobility, psychological distress, insomnia, and self-rated health. Our findings did support that vigorous activities mediated the relationship between LBP with psychological distress, insomnia and self-rated health. Our findings did not support vigorous activity as a mediator between LBP and mobility.

As it was documented in the literature vigorous activity may be beneficial for LBP, however, we have identified that it may also benefit other aspects of health related to back pain such as sleep, mental health and self-rated health. The findings from this study support that there are likely secondary benefits to engaging in vigorous activity among older adults with osteoarthritis and LBP. It is important to consider that the benefits of activity may take time to manifest in older adult populations (32). For example, an older adult may not feel pain relief or see weight loss changes or improved muscle strength until after participating in vigorous exercises long term.

Future directions for research include examining other sociocultural and psychological influences that are related to LBP and health outcomes. Factors such as pain catastrophizing, stigma, access to health care and biases and stereotypes may be related to LBP and mental health or physiological conditions. Information about these factors may lead to understanding of other treatment strategies that could be paired with vigorous exercise to better treat LBP and associated conditions. Utilizing vigorous exercise with a mental health treatment may be an effective way to address the disabling effects of LBP and related physical and mental outcomes. Researchers can further test interventions that include physical, psychological, social, and health-related quality-of-life domains in addition to vigorous activity treatments of pain given that all of these domains shape a person's pain experience based on the biopsychosocial model of pain.

### Clinical implications

This study showed further support for the relationships between LBP and co-occurring conditions. The results suggest that vigorous exercise may contribute to improved outcomes in the areas of mobility, psychological distress, insomnia, and self-rated health for persons with arthritis and LBP. Findings also highlight the burden of health conditions related to LBP, which may have implications for designing intervention studies for LBP. While prior studies have targeted back pain in isolation (42), it is important to treat the associated conditions as well including mobility limitations, psychological distress, and insomnia. Concomitant treatment of LBP and its comorbidities in older adults may better alleviate pain and improve quality of life and self-rated health. Unfortunately, to date, very few studies have examined interventions that treat both back pain and its associated conditions (43). Multidisciplinary-based rehabilitation should also be considered when treating LBP, given the psychological and physical factors associated with LBP (44).

It is unlikely that any one intervention will be uniformly beneficial for all older adults with LBP and associated conditions. Consequently, future research should focus on tailored interventions that may include individualized plans for each participant (41). These tailored intervention studies for adults with LBP should also have large sample sizes and target depression, fatigue/sleep, and mobility (45, 46). Future research is needed to identify effective ways to treat back pain and related conditions during times, such as the COVID-19 pandemic, when in-person visits and in-person physical therapies are less accessible for older adults with LBP (47). Self- management interventions that incorporate goal setting and tailored physical activities may be appropriate and sustainable for older adults living with back pain (48).

Clinicians play a key role in assisting patients with LBP in managing their pain and associated conditions. Understanding mechanisms that cause LBP may also be integral to determining the best treatment decisions for each patient. Translating current and future evidence into practice will allow clinicians to prescribe evidenced-based tailored interventions. Clinicians treating LBP should also check for the presence of associated conditions, including depression, insomnia, and mobility limitations, in order to develop a comprehensive treatment plan that will maximize the patient's quality of life. Due to the relationships that LBP has with psychological and psychosocial outcomes, incorporating a biopsychosocial approach into clinical practice may be an essential component for managing low back pain (49).

### Limitations and strengths

Several potential limitations were identified. First, the pain measure included in our study only assessed for the presence of pain over the preceding month. This measure does not capture chronic pain or provide in-depth description of the pain (e.g., dull and sharp). The measures for exercise and mobility limitations were coded as dichotomous variables, which is a potential limitation as well. Use of dichotomous coding for these two variables does not provide detail on the frequency or type of exercise or the severity of the mobility limitations. The findings should be interpreted with caution given the limitations of the outcome variables in the study. Furthermore, osteoarthritis in other joints such as the hip can contribute to or co-occur with back pain, and this mechanism should be further explored in older adults (50). It is possible that those with LBP were unable to engage in vigorous activity, in which case it would have no bearing on associated outcomes examined in this study. We did not account for medications the participants may have been taking, which is also a limitation. If the participants were taking medications for pain, insomnia, or to treat mental health conditions this may have influenced their outcomes in the study. Despite these potential limitations, the study has several strengths of note. The large, nationally representative sample of Medicare beneficiaries provides the ability to compare back pain to other common sites of musculoskeletal pain in older adults with arthritis. This nationally representative sample also enables generalization of findings to the U.S. older adult population.

Lastly, this study demonstrated that LBP over time is related to increased mobility limitations, psychological distress, insomnia, and poorer self-rated health. This highlights the important implications that LBP can have on quality of life and disability outcomes in aging adults (35).

## Conclusions

Pain, particularly in older adults, is a complex condition to treat, and not all pain can and/or should be treated with the same approach. Given the high prevalence of arthritis in older adults, the increased risk of disability if low back pain is simultaneously present, and the difficulty pinpointing the root cause of LBP, it is essential that researchers continue to examine what conditions are comorbid with LBP in this population as this may elucidate underlying pain mechanisms and suggest targets for intervention.

## Data availability statement

Publicly available datasets were analyzed in this study. This data can be found here: https://nhats.org/researcher/data-access.

## Ethics statement

The studies involving human participants were reviewed and approved by Johns Hopkins Internal Review Board. The patients/participants provided their written informed consent to participate in this study.

## Author contributions

JT and RS contributed to conception and design of the study. QL and ML conducted statistical analysis. JT and NR wrote the first draft of the manuscript. JT, NR, and QL wrote sections of the manuscript. SS assisted with edits and development of the manuscript. All authors contributed to manuscript revision, read, and approved the submitted version.

## Funding

JT was supported by the Robert Wood Johnson Harold. Amos Medical Faculty Program; NR was supported by the National Institute on Aging (K23AG058809); RS was supported by the NIH National Institute on Aging under Award (P01AG066603). QL was supported from the National Institute on Disability, Independent Living, and Rehabilitation Research (90RTGE0003). NIDILRR is a Center within the Administration for Community Living (ACL), Department of Health and Human Services (HHS). The contents of this publication do not necessarily represent the policy of NIDILRR, ACL, or HHS, and you should not assume endorsement by the Federal Government.

## Conflict of interest

The authors declare that the research was conducted in the absence of any commercial or financial relationships that could be construed as a potential conflict of interest.

## Publisher's note

All claims expressed in this article are solely those of the authors and do not necessarily represent those of their affiliated organizations, or those of the publisher, the editors and the reviewers. Any product that may be evaluated in this article, or claim that may be made by its manufacturer, is not guaranteed or endorsed by the publisher.
